# Serum proteomic, peptidomic and metabolomic profiles in myasthenia gravis patients during treatment with *Qiangji Jianli Fang*

**DOI:** 10.1186/1749-8546-7-16

**Published:** 2012-07-28

**Authors:** Chunmei Wang, Yonghai Lu, Zhixi Chen, Xiaobin Liu, Huangquan Lin, Hui Zhao, Jinyan Chen, Yiuwa Kwan, Saiming Ngai

**Affiliations:** 1 School of Life Sciences, The Chinese University of Hong Kong, Shatin, N.T, Hong Kong SAR, China; 2 Department of Nuclear Medicine, Guangzhou University of Chinese Medicine, Guangzhou, China; 3 School of Basic Medicine, Guangzhou University of Chinese Medicine, Guangzhou, China; 4 School of Biomedical Sciences, The Chinese University of Hong Kong, Hong Kong SAR, China

## Abstract

**Background:**

*Qiangji Jianli Fang* (QJF) has been used for treatment of myasthenia gravis (MG) in China. However, our understanding of the effects of QJF against MG at the molecular level is limited. This study aims to investigate the effects of QJF treatment of MG patients on the protein, peptide and metabolite levels in serum.

**Methods:**

High-throughput proteomic, peptidomic and metabolomic techniques were applied to investigate serum samples from 21 healthy individuals and 47 MG patients before and after QJF treatment via two-dimensional gel electrophoresis, matrix-assisted laser desorption/ionization time of flight mass spectrometry and liquid chromatography Fourier transform mass spectrometry, respectively.

**Results:**

After QJF treatment, the expression levels of peptides *m/z* 1865.019, 2021.128 and 1211.668 of complement C3f increased (*P* = 0.004, *P* = 0.001 and *P* = 0.043, respectively), while that of peptide *m/z* 1739.931 of component C4b decreased (*P* = 0.043), in the serum of MG patients. The levels of γ-aminobutyric acid (*P* = 0.000) and coenzyme Q4 (*P* = 0.000) resumed their normal states.

**Conclusion:**

QJF could inhibit the activity of the complement system and restore the normal levels of metabolites.

## Background

Myasthenia gravis (MG) is a chronic autoimmune neuromuscular disorder, with an incidence rate of 3–30/1,000,000 people per year [1]. MG patients can produce autoantibodies, such as anti-acetylcholine receptor antibody (AChRAb) and anti-muscle-specific receptor tyrosine kinase antibody (MuSKAb) through their own immune system to prevent muscle contraction, and cause muscle weakness and fatigue [2]. Genetic factors play an important role in MG [3]. In addition, infection with viruses or bacteria, such as poliovirus and *Escherichia coli*, may be involved in the pathogenesis of MG [4,5]. Anticholinesterase drugs, non-specific immunosuppressants, thymectomy and plasmapheresis are the main therapeutic approaches to MG [6-8]. However, the above treatments have some serious side effects, such as cardiac arrhythmia, osteoporosis and hypotension, and can not inhibit the relapse of patients’ symptoms and achieve complete remission [9]. Alternative treatments with higher efficacy and fewer side effects are required.

Chinese medicine (CM) has been practiced for many diseases, including cancer, cardiovascular disease, inflammation and Parkinson's disease, owing to its long-term efficacy and few side effects [10-14]. The mechanisms of CM immunomodulatory activity have previously been elucidated in several studies, revealing vital roles for immune effector cells, cytokine production and antibody production [15-18].

*Qiangji Jianli Fang* (QJF) is a CM prescription modified from the *Buzhong Yiqi* decoction including *Radix astragaliRadix codonopsis pilosulaeAtractylodes macrocephalaRadix angelicae sinensisCimicifugae rhizomaRadix bupleuriPericarpium citri reticulatae* and *Radix glycyrrhizae*[19]. Previous investigations have demonstrated that QJF exhibits similar efficiency in MG patients to prednisone, and plays long-term protective roles in MG by decreasing the AchRAb level and changing the expression of serum cytokines with fewer side effects [19-21]. Active components of the herbs in QJF, such as podocarpaside I in *Actaea podocarpa* and polysaccharide in *Angelica acutiloba kitagawa*, show characteristic effects on the complement system [22,23]. Herbs such as *R. bupleuri* and *R. glycyrrhizae* are beneficial for autoimmune diseases by inhibiting the production of serum autoantibodies and total IgG (Table 1). However, our understanding of the effects of QJF against MG at the molecular level is limited.

**Table 1:** Herbal components of QJF and their reported active ingredients with immunomodulatory effects

**Herbs**	**Active ingredients**	**References**
*R. astragali*	Polysaccharides; flavonoids; saponins	[24-26]
*R. codonopsis pilosulae*	Polysaccharide	[27,28]
*A. macrocephala*	Glycoprotein	[29,30]
*R. angelicae sinensis*	Sulfated polysaccharide	[31]
*C. rhizoma*	Cycloartane glycosides; cyclolanostane triterpene diglycosides	[32,33]
*R. bupleuri*	Bupleurum polysaccharides; saikosaponin	[34,35]
*Pericarpium citri reticulatae*	Synephrine	[36]
*R. glycyrrhizae*	Glycyrrhiza polysaccharide; liquiritigenin	[37,38]

Increased AChRAb and MuSKAb levels were detected in sera from MG patients by enzyme-linked immunosorbent assays [39]. Abnormal serum cytokine levels were also detected [40-42]. In addition, analyses of blood proteomic, peptidomic and metabolomic profiles have been employed to elucidate the pathological mechanisms of diseases and to evaluate the efficiency of drug treatments [43,44]. Differentially expressed proteins, peptides and metabolites in serum from MG patients have been compared with those in healthy people by two-dimensional gel electrophoresis (2-DE) and mass spectrometry [45-47]. However, no reports have shown the changes in the serum proteomic, peptidomic and metabolomic patterns after QJF treatment.

In this study, high-throughput proteomic, peptidomic and metabolomic techniques were adopted to elucidate the effects of QJF on MG patients using matrix-assisted laser desorption/ionization time-of-flight mass spectrometry (MALDI-TOF MS), 2-DE and liquid chromatography Fourier transform mass spectrometry (LC-FTMS), respectively.

## Methods

### Herbs

QJF was produced by the First Affiliated Hospital of Guangzhou University of Chinese Medicine from a boiled water extraction of eight herb components as follows: 60 g of *R. astragali*, 30 g of *R. codonopsis pilosulae*, 15 g of *A. macrocephala*, 10 g of *R. angelicae sinensis*, 10 g of *C. rhizoma*, 10 g of *R. bupleuri*, 5 g of *P. citri reticulatae* and 5 g of *R. glycyrrhizae*. Compared with the herb levels in the *Buzhong Yiqi* decoction, QJF contained decreased levels of *P. citri reticulatae* (5 g vs. 6 g) and *R. glycyrrhizae* (5 g vs. 9 g *R. glycyrrhizae preparata*) and increased levels of *R. astragali* (60 g vs. 18 g), *R. codonopsis pilosulae* (30 g vs. 6 g *Panax ginseng*), *A. macrocephala* (15 g vs. 9 g), *R. angelicae sinensis* (10 g vs. 3 g), *C. rhizoma* (10 g vs. 6 g) and *R. bupleuri* (10 g vs. 6 g). The herbs were purchased from Zhixin Medicine Health Co. Ltd. (China) and identified by the School of Chinese Pharmaceutical Science, Guangzhou University of Chinese Medicine, China. For the extraction, the herbs were boiled in four volumes of water for 1 h, and the extraction step was repeated once. The two extracts were mixed together for use.

### Participants

This study was approved by Ethics Commitee of Guangzhou University of Chinese Medicine, and all participants provided informed consent according to institutional guidelines. Peripheral blood samples were obtained from 47 patients (18 males and 29 females; average age: 40 years) diagnosed with MG in the First Affiliated Hospital of Guangzhou University of Chinese Medicine. All MG patients were confirmed by the neostigmine test. According to the Osserman classification [48], 4 patients were class I, 34 patients were class II (17 class IIa; 17 class IIb), 4 patients were class IV and 5 patients were class V. All the patients were treated with QJF orally once per day for 2 months. Peripheral blood samples from 21 healthy individuals (10 males and 11 females; average age: 34 years) were collected as controls.

### Preparation of serum samples

Before and after the 2-month treatment, blood samples were collected, allowed to clot at 4 °C overnight and centrifuged at 1,000 × *g* for 10 min. The sera were collected and frozen in aliquots for storage at −80 °C until analysis.

### Albumin/IgG depletion

A Qproteome Albumin/IgG Depletion Kit (Qiagen, USA) was used for depletion of albumin and IgG from the serum samples according to the manufacturer’s instructions. Briefly, an aliquot of serum (50 μL) was applied to a depletion spin column and the eluate was collected by centrifugation at 500 × *g* for 10 s. The samples were desalted by acetone (Merck, Germany) precipitation and resuspended in buffer comprising 7 M urea (GE Healthcare, Sweden), 2 M thiourea (GE Healthcare, UK), 2% (w/v) 3-[(3-cholamidopropyl)-dimethylammonio]-1-propanesulfonate (CHAPS) (USB, Germany) and 1% (w/v) dithiothreitol (USB, Canada). Subsequently, the samples were quantified using a PlusOne™ 2D Quant Kit (GE Healthcare, Sweden) according to the manufacturer’s instructions.

### Two-dimensional gel elctrophoresis

2-DE experiments were performed following the protocol of GE Healthcare Life Science with some modifications. Albumin/IgG-depleted sera (100 μg) were loaded onto immobilized pH strips (pH 3–10) of 13 cm in length. Isoelectric focusing was performed using an Ettan™ IPGphor™ Isoelectric Focusing System (GE Healthcare, Sweden) with a total voltage hours (Vhs) of 38890 Vhs. The IPG strips were sequentially equilibrated in buffer (50 mM Tris–HCl, pH 8.8, 6 M urea, 30% (v/v) glycerol, 2% (w/v) SDS, 0.002% (w/v) bromophenol blue) containing dithiothreitol (10 mg/mL) and iodoacetamide (25 mg/mL; GE Healthcare, UK) for 15 min before separation by 11% sodium dodecyl sulfate polyacrylamide gel electrophoresis. The gels were silver-stained and analyzed using ImageMaster™ 2D Platinum ver. 5.0 software (GE Healthcare, Sweden). The experiment was repeated three times. Differentially expressed protein spots (≥ 1.5-fold) were excised. After in-gel digestion with 20 ng/μL trypsin (Promega, USA), the peptides were extracted with 2.5% trifluoroacetic acid (Sigma, Germany) and 80% acetonitrile (ACN) (Merck, Germany) for mass spectrometry.

### Peptide capture

Peptides in serum were extracted using Dynabeads RPC 18 (Invitrogen Dynal AS, Norway) according to the manufacturer’s instructions. Serum (50 μL) was added to a vial containing 20 μL (0.25 mg) of Dynabeads and incubated at room temperature for 5 min. Peptides were eluted with 10 μL of 80% ACN for mass spectrometry.

### Mass spectrometry

Peptide solution (1.5 μL) extracted from a 2D gel spot or captured from serum was spotted onto a MALDI target plate, followed by spotting of 0.5 μL of α-cyano-4-hydroxy-cinnamic acid (Sigma, USA). Peptides with the mass range *m/z* 700–4000 were detected using a 4700 MALDI-TOF/TOF mass spectrometer (Applied Biosystems, USA) in the reflectron positive-ion mode and accumulated from 2000 laser shots with acceleration of 20 kV. The MS spectra were internally calibrated using porcine trypsin autolytic products (*m/z* 842.509, 1045.564, 1940.935 and 2211.104). The MS peaks (MH^+^) were detected with a minimum S/N ratio of ≥ 20 and a cluster area S/N threshold of ≥ 25 without smoothing or raw spectrum filtering. Peptide precursor ions corresponding to contaminants, including keratin and the trypsin autolytic products, were excluded with a mass tolerance of ± 0.2 Da. The filtered precursor ions with a defined threshold (S/N ratio ≥ 50) were selected for the MS/MS scan. Fragmentation of precursor ions was performed using the MS/MS 1-kV positive mode with collision-induced dissociation on and argon as the collision gas. MS/MS spectra were accumulated from 3000 laser shots using default calibration with Glu-Fibrinopeptide B (Applied Biosystems, USA). The MS/MS peaks were detected for a minimum S/N ratio of ≥ 3 and a cluster area S/N threshold of ≥ 15 with smoothing.

### Metabolomic analysis

Serum was transferred into an Eppendorf tube and diluted three-fold (v/v) with ACN. The mixture was shaken vigorously for 30 s. After centrifugation at 9,600 × *g* for 10 min at room temperature, the supernatant was analyzed using a Dionex Ultimate 3000 2D Nanoflow LC System (Bruker Daltonics Inc., USA) coupled to an Apex Ultra 7.0 Hybrid Qh-FTMS (Bruker Daltonics Inc., USA) equipped with an electrospray ionization source. An Atlantis T3 3 μm column (2.1 mm i.d. × 150 mm; Waters, USA) was used. The column was maintained at 35 °C. The mobile phases A and B were water with 0.1% formic acid (Sigma, USA) and ACN with 0.1% formic acid, respectively. The gradient duration program was: 0–2 min, 5% B; 2–17 min, 5–95% B; 17–20 min, 95% B; and 20–21 min, 95–5% B. The flow rate was 0.2 mL/min. The spectra were acquired over the *m/z* 50–1000 range in the positive ion mode. The capillary voltage and spray shield were set to 4200 and 3500 V, respectively. The dry gas was set to 6 L/min at a temperature of 200 °C. The neb gas was set to 3 L/min.

### Data processing

The MS and MS/MS data acquired by MALDI-TOF MS were loaded into GPS Explorer™ software ver. 3.5 (Applied Biosystems, USA) and searched against the NCBInr human database using the Mascot search engine (Matrix Science, UK) for protein and peptide identification. The following search parameters were used: monoisotopic peptide mass (MH^+^); mass range, 700–4000 Da; one missed cleavage per peptide; enzyme, trypsin (no enzyme was selected for serum peptide data searching); taxonomy, human; pI, 0–14; precursor ion mass tolerance, 50 ppm; MS/MS fragment ion mass tolerance, 0.1 Da; and variable modifications, oxidation for methionine (no modification was selected for serum peptide data searching). Known contaminant ions corresponding to keratin and/or trypsin were excluded from the peak lists before database searching. Protein score was calculated automatically by Mascot search engine based on the comparison of peptide masses and peptide fragment ion masses to amino acid sequences in database. The top ten hits for each protein search were reported. Proteins with MOWSE scores above 70 and at least four matched peptides were accepted as identified. The spectra of the captured serum peptides were imported to Markerview™ software ver. 1.2.0.6 (Applied Biosystems/MDS SCIEX, USA) using the following spectra processing options: mass tolerance, 50 ppm; minimum required response, 200; and maximum number of peaks, 20000.

LC-FTMS data were exported by Data Analysis ver. 4.0 software (Bruker Daltonics Inc., USA) and analyzed by SIMCA-P ver. 12.0 software (Umetrics AB, Sweden). The data were presented as the mean ± standard deviation (SD). The significance of differences was evaluated by Student's *t*-test using SPSS 13.0 for Windows software (SPSS Inc., USA). *P* values less than 0.05 were considered statistically significant.

## Results and discussion

In this study, three approaches were applied to investigate the changes in the serum profiles of MG patients during QJF treatment. The proteomic approach detects serum proteins of > 20 kDa, the peptidomic approach evaluates peptides with a mass range of 700–4000 Da and the metabolomic approach investigates metabolites of < 1000 Da. Although serum proteins, peptides and metabolites can serve as biomarkers to indicate the progression of a diseased state, changes in the processed products, such as peptides and metabolites, can be more complementary to a certain diseased state and drug treatment.

### Serum proteomic profiles

Arbitrarily combined serum samples from healthy controls and MG patients before and after QJF treatment were prepared, respectively. Before the proteomic analysis, most of the highly abundant proteins, such as albumin and IgG, were depleted from the serum samples. 2-DE and MALDI-TOF MS were performed to identify differentially expressed proteins in the serum samples from the different groups.

**Figure 1:**
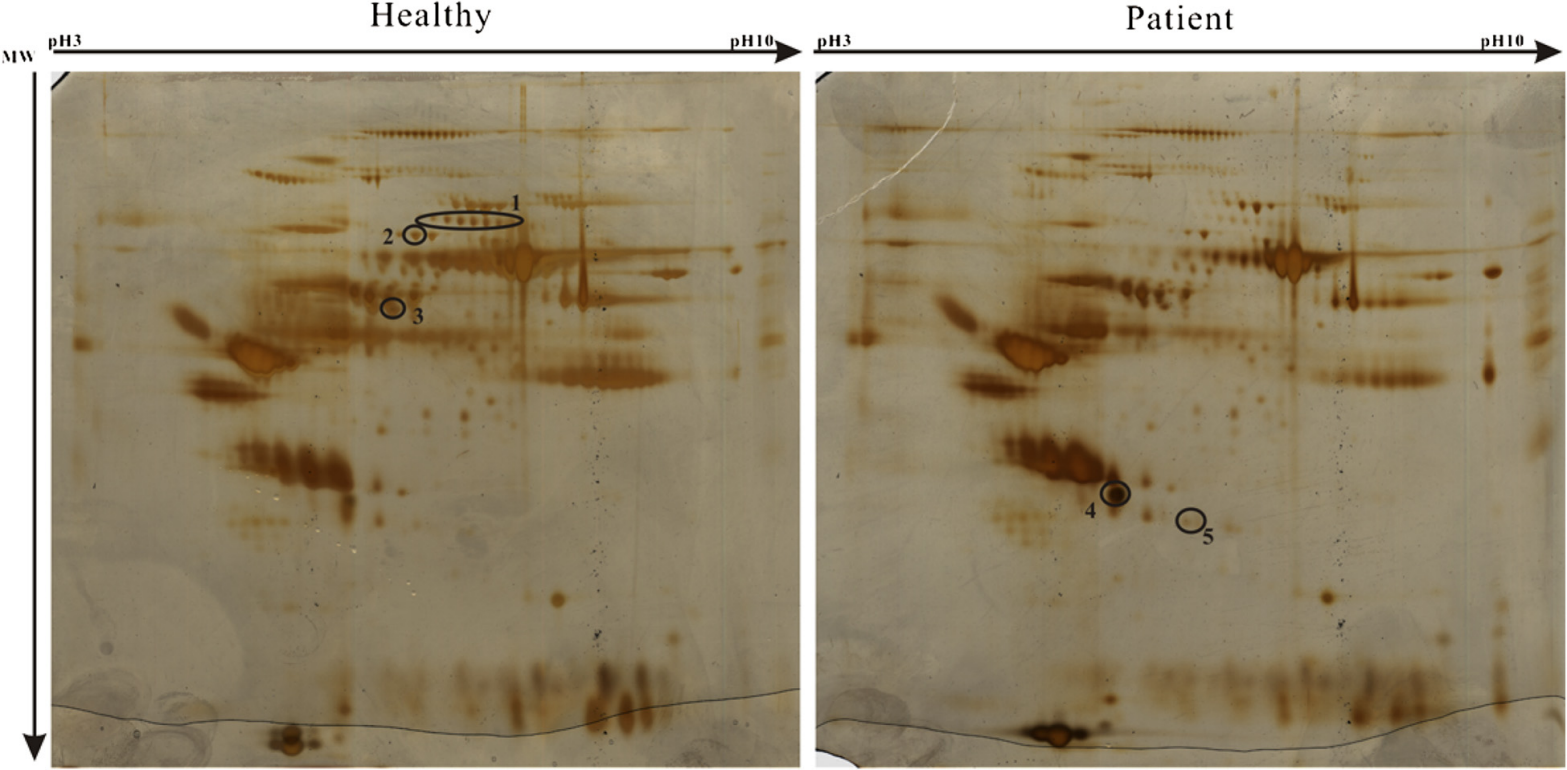
**2-DE gel patterns derived from healthy control and MG patient sera.** Aliquots containing 100 μg of albumin/IgG-depleted proteins extracted from the sera of healthy controls (left) and MG patients (right) were subjected to isoelectric focusing (total: 38890 Vhs) using 13-cm strips (pH 3–10). The 2-DE gels were silver-stained. Differentially expressed proteins (> 1.5-fold) were labeled in the gels. The fold changes were calculated from three replicates. The X-axis shows pI 3–10 and the Y-axis shows the molecular weight in kDa.

In the 2-DE gels, ten differentially expressed spots were detected in the serum of MG patients compared with the healthy controls (Figure 1). Five proteins were identified in these ten spots through MALDI-TOF MS and NCBI database searches. Among them, α-2-macroglobulin, gelsolin and hemopexin were downregulated (decreases of 2.49-, 1.91- and 2.07-fold with *P* = 0.002, 0.046 and 0.011, respectively) while haptoglobin and haptoglobin-related protein were upregulated (increases of 1.58- and 2.62-fold with *P* = 0.062 and 0.005, respectively) in the serum of MG patients compared with healthy controls (Table 2), suggesting immunoreactivity in the MG patients. In the serum of ankylosing spondylitis patients, another autoimmune disease, haptoglobin and its precursor were reported to show significant increases [49,50]. The similar results observed in MG patients in the present study further confirm their roles in the pathological process of autoimmune diseases. α-2-macroglobulin is a carrier protein for hormones and an inhibitor of proteolytic enzyme activities [51], and its decrease induces abnormalities in protease metabolism in MG patients. The decrease in hemopexin in the serum of MG patients may reflect an anemic state during the chronic process [52].

**Table 2:** Differentially expressed proteins in the serum profiles of MG patients

**Spot**	**Protein name**	**Accession Number (Swiss-Prot)**	**No. of matched peptides**	**Protein score**	**Fold change**^**a**^	***P*value**^**b**^
1	α-2-macroglobulin	P01023	17	207	−2.49	0.002
2	Gelsolin	P06396	10	90	−1.91	0.046
3	Hemopexin	P02790	13	180	−2.07	0.011
4	Haptoglobin	P00738	12	134	1.58	0.062
5	Haptoglobin-related protein	P00739	10	148	2.62	0.005

^a^ The ratio of the volume percentage (vol %) between MG patient and healthy control.

^b^*P* values were calculated by Student’s *t*-test.

To evaluate the effects of QJF on MG patients, the serum proteomic profiles of MG patients before and after treatment were compared using the above method. However, no differentially expressed proteins were identified, even in the five proteins listed above, indicating that QJF cannot reverse the expression changes in these proteins.

### Serum peptidomic profiles

The serum peptides of all samples were extracted using Dynabeads and analyzed by MALDI-TOF MS. Around 278 peptides within the mass range of *m/z* 700–4000 were extracted from the raw spectra using the spectra processing options described in the Methods section. Through MarkerView™ ver. 1.2 software analysis, 16 peptides identified as six proteins showed significant decreases (*P* < 0.05) in MG patients and seven peptides changed their quantities after QJF treatment (*P* < 0.05) (Table 3).

**Table 3:** Differentially expressed peptides in the serum profiles of MG patients before and after QJF treatment

**Protein name**	**Peptide mass**	**Sequence**	**Patient (P) vs. healthy control(C)**			**After (A) vs. Before (B) treatment**		
			**Change**	***P*value**	**Fold change (C/P)**	**Change**	***P*value**	**Fold change (B/A)**
Complement C3f	1777.922	SKITHRIHWESASLL	Down	0.000	1.808	-	-	-
	1865.019	SSKITHRIHWESASLL	Down	0.000	2.311	Up	0.004	0.582
	1934.125	SKITHRIHWESASLLR	Down	0.043	1.412	-	-	-
	2021.128	SSKITHRIHWESASLLR	Down	0.000	2.488	Up	0.001	0.364
	1211.668	RIHWESASLL	-	-	-	Up	0.043	0.753
Component C4b	1625.975	NGFKSHALQLNNRQ	Down	0.010	5.421	-	-	-
	1739.931	NGFKSHALQLNNRQI	Down	0.013	2.295	Down	0.043	1.567
Kallidin II	904.682	RPPGFSPF	Down	0.003	1.442	Down	0.000	3.698
α-fibrinogen precursor	905.452	FLAEGGGVR	Down	0.020	2.625	-	-	-
	1020.516	DFLAEGGGVR	Down	0.001	3.435	Up	0.000	0.381
	1077.550	GDFLAEGGGVR	Down	0.001	2.156	-	-	-
	1206.605	EGDFLAEGGGVR	Down	0.023	4.059	-	-	-
	1350.655	SGEGDFLAEGGGVR	Down	0.019	2.284	-	-	-
	1465.701	DSGEGDFLAEGGGVR	Down	0.041	3.008	-	-	-
	2931.280	SSSYSKQFTSSTSYNRGDSTFESKSY	Down	0.006	2.025	-	-	-
	2553.160	SSSYSKQFTSSTSYNRGDSTFES	-	-	-	Down	0.005	1.409
Fibrinoligase	2602.337	AVPPNNSNAAEDDLPTVELQGVVPR	Down	0.043	1.824	-	-	-
Prothrombin	1389.686	TSEYQTFFNPR	Down	0.044	1.675	-	-	-

Note: “-”indicates that the peptide showed no differential expression.

The expression levels of complement C3f peptides, such as *m/z* 1777.922, 1865.019, 1934.125 and 2021.128, were significantly decreased in MG patients, while the expression of complement C3f peptides *m/z* 1865.019, 2021.128 and 1211.668 were increased after QJF treatment (Table 3). Complement C3f is the released inactive peptide from component C3 [53], suggesting that QJF could increase the degradation of C3 and alleviate the high level of this complement component in the blood of MG patients.

The complement C3 level is correlated with the clinical severity of AChRAb-positive generalized MG [54]. Suppressed anti-AChR IgG production and deposition of attack complexes at the endplates were found in C3 (−/−) mice, which showed resistance to MG [55]. Therefore, QJF could be beneficial to MG patients via degradation of complement component C3.

Peptide *m/z* 1739.931 of complement C4b decreased after QJF treatment (Table 3). C4b is a part of C3-convertase and takes part in C3 activation, which influences the production of active C3b [56]. The mechanism of the QJF effect on the immunoreactivity could involve an increase in the degradation of complement C3 and a decrease in the production of the active complement C3 fragment. These findings suggest that inhibition of complement components is a potential therapeutic strategy with high efficacy for autoimmune diseases, including MG [7,57].

Kallidin II was decreased in MG patients (decrease of 1.442-fold compared with healthy controls), and remained decreased even after QJF treatment (decrease of 3.698-fold compared with untreated MG patients) (*P* = 0.000) (Table 3). Kallidin is a pro-inflammatory kinin peptide that acts as a stimulant for several inflammatory cytokines, such as tumor necrosis factor, interleukin-1 and interleukin-6, and is involved in many physiological and pathological processes [58]. Abnormal serum cytokine levels were shown to be related to the pathogenesis of MG [40]. The decrease in kallidin II after QJF treatment may inhibit the stimulation of cytokines and reduce the activity of inflammatory molecules in MG patients.

Peptides belonging to α-fibrinogen precursor, fibrinoligase and prothrombin were also detected in this study. These proteins are involved in blood coagulation. Upon QJF treatment, most of the peptides showed no change, except for the increase in peptide *m/z* 1020.516 (*P* = 0.000) and the decrease in peptide *m/z* 2553.160 (*P* = 0.005). However, no studies have shown their associations with MG.

### Serum metabolomic profiles

In our previous study [47], nine differentially expressed metabolites were identified in the serum of MG patients, including γ-aminobutyric acid (GABA), coenzyme Q4, pipecolic acid, 5,8-tetradecadienoic acid, sphingosine-1-phosphate, bisnorcholic acid, chenodeoxycholylglycine, coprocholic acid and cholylglycine. In the present study, we compared the serum metabolic profiles in MG patients before and after QJF treatment by LC-FTMS. Eight metabolites with significant changes in abundance after QJF treatment were identified (Table 4). The results showed that the GABA level was increased after QJF treatment. GABA has an inhibitory role in autoimmune inflammation, and an inefficient GABA signaling system may result in unchecked proinflammatory cytokine production via the p38 MAPK pathway [59,60]. Therefore, QJF could improve the deficiency status of GABA in the serum of MG patients.

**Table 4:** Serum metabolite profiles of MG patients before and after QJF treatment

**Metabolite**	**Formula**	**± ppm**^**a**^	**Peak area (Mean ± SD**^**b**^**)**		**% Change**^**c**^	***P*value**^**d**^
			**Before therapy**	**After therapy**		
γ-aminobutyric acid	C_4_H_9_NO_2_	−71.7	10.2 ± 0.52	15.80 ± 0.81	54.9	0.000
Coenzyme Q4	C_29_H_42_O_4_	43.1	12.28 ± 3.41	7.89 ± 0.89	−35.7	0.000
Taurallocholic acid	C_26_H_45_NO_7_S	−75.8	0.03 ± 0.17	6.72 ± 5.32	19016.7	0.000
Dipalmitoylphosphatidic acid	C_35_H_69_O_8_P	92.9	0.19 ± 0.38	6.65 ± 2.35	3397.8	0.000
Phytosphingosine	C_18_H_39_NO_3_	−2.3	1.73 ± 1.53	19.11 ± 7.29	1004.8	0.000
5b-Cyprinolsulfate	C_27_H_48_O_8_S	−50.2	0.38 ± 0.61	2.63 ± 2.01	591.9	0.000
Thromboxane B2	C_20_H_34_O_6_	40	1.13 ± 1.45	2.92 ± 1.91	158.3	0.000
Biliverdin IX	C_33_H_34_N_4_O_6_	3.6	22.71 ± 5.78	6.36 ± 3.54	−71.9	0.000

^a^Mass differences between the calculated and measured metabolite masses.

^b^Mean peak areas of metabolites in patients before and after QJF treatment. SD denotes standard deviation.

^c^Changes are calculated as the difference in the median concentrations before and after QJF treatment.

^d^*P* values were calculated by Student’s *t*-test.

Coenzyme Q4 was a detected metabolite with decreased expression after QJF treatment. Coenzyme Q4 is a member of the ubiquinone family. Ubiquinone and ubiquinol have protective effects on serum low-density lipoprotein from lipid peroxidation [61]. Another ubiquinone coenzyme, Q10, exerts anti-inflammatory properties via NF-κB1-dependent gene expression [62]. However, no reports have suggested a function for coenzyme Q4 in serum, and the issue of whether it exerts anti-inflammatory properties requires further investigation.

In addition to the above two metabolites, six other metabolites also changed their quantities following QJF treatment, although no changes were detected in MG patients compared with normal healthy controls. Among these metabolites, phytosphingosine and dipalmitoylphosphatidic acid have anti-inflammatory effects [63,64]. The increased level of thromboxane B2, an inactive product of thromboxane A2, may improve the high coagulation state of MG patients, while the decrease in biliverdin IX indicated less hemoglobin breakdown. Interestingly, increases in taurallocholic acid and 5b-cyprinolsulfate, two bile acids involved in fat processing, were also detected. However, no studies have reported their functions in autoimmune or inflammatory diseases.

## Conclusion

QJF could inhibit the activity of the complement system and resume the normal levels of metabolites in MG patients. The findings of the present and previous studies suggest that QJF is an effective drug for treatment of MG.

## Abbreviations

QJF, Qiangji Jianli Fang; MG, Myasthenia gravis; AChRAb, Anti-acetylcholine receptor antibody; MuSKAb, Anti-muscle-specific receptor tyrosine kinase antibody; CM, Chinese medicine; 2-DE, Two-dimensional gel electrophoresis; MALDI-TOF MS, Matrix-assisted laser desorption/ionization time-of-flight mass spectrometry; LC-FTMS, Liquid chromatography Fourier transform mass spectrometry; CHAPS, 3-[(3-cholamidopropyl)-dimethylammonio]-1-propanesulfonate; Vhs, Voltage hours; ACN, Acetonitrile; GABA, γ-aminobutyric acid.

## Competing interests

The authors declare that they have no competing interests.

## Authors' contributions

CW, YL, ZC and SN designed the study. CW and YL conducted the experiments and contributed equally to the study. CW, YL, YK and SN wrote the manuscript. ZC, XL, HQ, HZ and JC recruited patients, performed the treatment and collected the samples. All authors read and approved the final version of the manuscript.

## References

[B1] McGrogan A Sneddon S de Vries CS The incidence of myasthenia gravis: a systematic literature review Neuroepidemiology 2010 34 171 183 20130418 10.1159/000279334

[B2] Gomez AM Van Den Broeck J Vrolix K Janssen SP Lemmens MA Van Der Esch E Duimel H Frederik P Molenaar PC Martínez-Martínez P De Baets MH Losen M Antibody effector mechanisms in myasthenia gravis-pathogenesis at the neuromuscular junction Autoimmunity 2010 43 353 370 20380584 10.3109/08916930903555943

[B3] Giraud M Vandiedonck C Garchon HJ Genetic factors in autoimmune myasthenia gravis Ann N Y Acad Sci 2008 1132 180 192 18567868 10.1196/annals.1405.027

[B4] Cavalcante P Barberis M Cannone M Baggi F Antozzi C Maggi L Cornelio F Barbi M Dido P Berrih-Aknin S Mantegazza R Bernasconi P Detection of poliovirus-infected macrophages in thymus of patients with myasthenia gravis Neurology 2010 74 1118 1126 20368632 10.1212/WNL.0b013e3181d7d884

[B5] Stefansson K Dieperink ME Richman DP Marton LS Sharing of epitopes by bacteria and the nicotinic acetylcholine receptor: a possible role in the pathogenesis of myasthenia gravis Ann N Y Acad Sci 1987 505 451 460 2446557 10.1111/j.1749-6632.1987.tb51315.x

[B6] Mantegazza R Bonanno S Camera G Antozzi C Current and emerging therapies for the treatment of myasthenia gravis Neuropsychiatric Disease and Treatment 2011 7 151 160 21552317 10.2147/NDT.S8915 PMC3083988

[B7] Tüzün E Huda R Christadoss P Complement and cytokine based therapeutic strategies in myasthenia gravis J Autoimmun 2011 37 136 143 21636248 10.1016/j.jaut.2011.05.006

[B8] Chien PJ Yeh JH Chiu HC Hsueh YM Chen CT Chen MC Shih CM Inhibition of peripheral blood natural killer cell cytotoxicity in patients with myasthenia gravis treated with plasmapheresis Eur J Neurol 2011 18 1350 1357 21554496 10.1111/j.1468-1331.2011.03424.x

[B9] García-Carrasco M Escarcega RO Fuentes-Alexandro S Riebeling C Cervera R Therapeutic options in autoimmune myasthenia gravis Autoimmun Rev 2007 6 373 378 17537383 10.1016/j.autrev.2007.01.001

[B10] Lin H Liu J Zhang Y Developments in cancer prevention and treatment using traditional Chinese medicine Frontiers of Medicine 2011 5 127 133 21695616 10.1007/s11684-011-0137-7

[B11] Liu ZL Liu JP Zhang AL Wu Q Ruan Y Lewith G Visconte D Chinese herbal medicines for hypercholesterolemia Cochrane Database Syst Rev 2011 7 CD008305 21735427 10.1002/14651858.CD008305.pub2 PMC3402023

[B12] He DY Dai SM Anti-inflammatory and immunomodulatory effects of paeonia lactiflora pall., a traditional chinese herbal medicine Frontiers in Pharmacology 2011 2 10 21687505 10.3389/fphar.2011.00010 PMC3108611

[B13] Sun Y Zang Z Xu X Zhang Z Zhong L Zan W Zhao Y Sun L Experimental investigation of the immunoregulatory and anti-inflammatory effects of the traditional Chinese medicine "Li-Yan Zhi-Ke Granule" for relieving chronic pharyngitis in rats Mol Biol Rep 2011 38 199 203 20349277 10.1007/s11033-010-0095-1

[B14] Pan W Kwak S Liu Y Sun Y Fang Z Qin B Yamamoto Y Traditional chinese medicine improves activities of daily living in Parkinson's disease Parkinson's Disease 2011 2011 789506 10.4061/2011/789506 PMC3109418 21687764

[B15] Ho LJ Lai JH Chinese herbs as immunomodulators and potential disease-modifying antirheumatic drugs in autoimmune disorders Curr Drug Metab 2004 5 181 192 15078195 10.2174/1389200043489081

[B16] Lai JH Immunomodulatory effects and mechanisms of plant alkaloid tetrandrine in autoimmune diseases Acta Pharmacol Sin 2002 23 1093 1101 12466046

[B17] Chen ZJ Li YS Li YK Review of study on mechanism of traditional Chinese medicine in treating autoimmunity disease Journal of Chinese Medicinal Materials 2003 26 218 221 12856473

[B18] Huang CF Lin SS Liao PH Young SC Yang CC The immunopharmaceutical effects and mechanisms of herb medicine Cell Mol Immunol 2008 5 23 31 18318991 10.1038/cmi.2008.3 PMC4652916

[B19] Chen XK Wang LF Zhao H Wu YH Chen ZX Effect of Qiangji Jianli Fang on the serum levels of cytokines (IL-2, TNF, IL-6) in rat models of spleen-kidney deficiency syndrome Journal of Radioimmunology 2010 23 282 284

[B20] Liu XB Deng GZ Clinical investigation of Qiangji JianLi Fang on myasthenia gravis patients with spleen-kidney deficiency syndrome Traditional Chinese Drug Research & Clinical Pharmacology 2004 15 361

[B21] Deng TT Li RX Li SHM Zhang SHP Liu XB Yang WH Deng GZ Curative effect of Qiang J Jian L capsule in a randomized, double-blind, self-cross-controlled test for myasthenia gravis Journal of Guangzhou University of Traditional Chinese Medicine 1992 9 7 10

[B22] Ali Z Khan SI Khan IA New cycloartane-type triterpene arabinosides from the roots of Actaea podocarpa and their biological study Planta Med 2007 73 699 703 17562492 10.1055/s-2007-981533

[B23] Yamada H Kiyohara H Cyong JC Otsuka Y Studies on polysaccharides from Angelica acutiloba--IV. Characterization of an anti-complementary arabinogalactan from the roots of Angelica acutiloba Kitagawa Mol Immunol 1985 22 295 304 4000133 10.1016/0161-5890(85)90165-8

[B24] Jiang JB Qiu JD Yang LH He JP Smith GW Li HQ Therapeutic effects of astragalus polysaccharides on inflammation and synovial apoptosis in rats with adjuvant-induced arthritis Int J Rheum Dis 2010 13 396 405 21199477 10.1111/j.1756-185X.2010.01555.x

[B25] Liu J Hu X Yang Q Yu Z Zhao Z Yi T Chen H Comparison of the immunoregulatory function of different constituents in radix astragali and radix hedysari J Biomed Biotechnol 2010 2010 479426 20224658 10.1155/2010/479426 PMC2836181

[B26] Zhao P Su G Xiao X Hao E Zhu X Ren J Chinese medicinal herb Radix Astragali suppresses cardiac contractile dysfunction and inflammation in a rat model of autoimmune myocarditis Toxicol Lett 2008 182 29 35 18782607 10.1016/j.toxlet.2008.08.002

[B27] Sun YX Immunological adjuvant effect of a water-soluble polysaccharide, CPP, from the roots of Codonopsis pilosula on the immune responses to ovalbumin in mice Chem Biodivers 2009 6 890 896 19551730 10.1002/cbdv.200800154

[B28] Wang ZT Ng TB Yeung HW Xu GJ Immunomodulatory effect of a polysaccharide-enriched preparation of Codonopsis pilosula roots Gen Pharmacol 1996 27 1347 1350 9304404 10.1016/s0306-3623(96)00084-5

[B29] Kim SH Jung HN Lee KY Kim J Lee JC Jang YS Suppression of Th2-type immune response-mediated allergic diarrhea following oral administration of traditional Korean medicine: Atractylodes macrocephala Koidz Immunopharmacol Immunotoxicol 2005 27 331 343 16114514 10.1081/iph-200067950

[B30] Lee JC Lee KY Son YO Choi KC Kim J Kim SH Chung GH Jang YS Stimulating effects on mouse splenocytes of glycoproteins from the herbal medicine Atractylodes macrocephala Koidz Phytomedicine 2007 14 390 395 17084605 10.1016/j.phymed.2006.09.012

[B31] Yang T Jia M Zhou S Pan F Mei Q Antivirus and immune enhancement activities of sulfated polysaccharide from Angelica sinensis Int J Biol Macromol 2012 50 768 772 22155400 10.1016/j.ijbiomac.2011.11.027

[B32] Nishida M Yoshimitsu H Nohara T Three cycloartane glycosides from Cimicifuga rhizome and their immunosuppressive activities in mouse allogeneic mixed lymphocyte reaction Chem Pharm Bull(Tokyo) 2003 51 354 356 12612431 10.1248/cpb.51.354

[B33] Pan RL Chen DH Si JY Zhao XH Li Z Cao L Immunosuppressive effects of new cyclolanostane triterpene diglycosides from the aerial part of Cimicifuga foetida Arch Pharm Res 2009 32 185 190 19280146 10.1007/s12272-009-1133-1

[B34] Wang Z Li H Xu H Yue XL Cheng XQ Hou WJ Zhang YY Chen DF Beneficial effect of Bupleurum polysaccharides on autoimmune disease induced by Campylobacter jejuni in BALB/c mice J Ethnopharmacol 2009 124 481 487 19467314 10.1016/j.jep.2009.05.013

[B35] Ushio Y Oda Y Abe H Effect of saikosaponin on the immune responses in mice Int J Immunopharmacol 1991 13 501 508 1783462 10.1016/0192-0561(91)90069-j

[B36] Shi Q Liu Z Yang Y Geng P Zhu YY Zhang Q Bai F Bai G Identification of anti-asthmatic compounds in Pericarpium citri reticulatae and evaluation of their synergistic effects Acta Pharmacol Sin 2009 30 567 575 19363516 10.1038/aps.2009.36 PMC4002823

[B37] Yang G Yu Y Immunopotentiating effect of traditional Chinese drugs–ginsenoside and glycyrrhiza polysaccharide Proc Chin Acad Med Sci Peking Union Med Coll 1990 5 188 193 2293226

[B38] Lee JY Lee JH Park JH Kim SY Choi JY Lee SH Kim YS Kang SS Jang EC Han Y Liquiritigenin, a licorice flavonoid, helps mice resist disseminated candidiasis due to Candida albicans by Th1 immune response, whereas liquiritin, its glycoside form, does not Int Immunopharmacol 2009 9 632 638 19264152 10.1016/j.intimp.2009.02.007

[B39] Keesey J Aarli J Something in the Blood? A history of the autoimmune hypothesis regarding myasthenia gravis J Hist Neurosci 2007 16 395 412 17966056 10.1080/09647040600675322

[B40] Yeh JH Wang SH Chien PJ Shih CM Chiu HC Changes in serum cytokine levels during plasmapheresis in patients with myasthenia gravis Eur J Neurol 2009 16 1318 1322 19614971 10.1111/j.1468-1331.2009.02729.x

[B41] Na SJ So SH Lee KO Choi YC Elevated serum level of interleukin-32α in the patients with myasthenia gravis J Neurol 2011 258 1865 1870 21487807 10.1007/s00415-011-6036-7

[B42] Kim JY Yang Y Moon JS Lee EY So SH Lee HS Park KD Choi YC Serum BAFF expression in patients with myasthenia gravis J Neuroimmunol 2008 199 151 154 18586330 10.1016/j.jneuroim.2008.05.010

[B43] Al-Mubarak R Vander Heiden J Broeckling CD Balagon M Brennan PJ Vissa VD Serum metabolomics reveals higher levels of polyunsaturated Fatty acids in lepromatous leprosy: potential markers for susceptibility and pathogenesis PLoS Negl Trop Dis 2011 5 e1303 21909445 10.1371/journal.pntd.0001303 PMC3167790

[B44] Hortin GL The MALDI-TOF mass spectrometric view of the plasma proteome and peptidome Clin Chem 2006 52 1223 1237 16644871 10.1373/clinchem.2006.069252

[B45] Liu P Zhang YY Qiao J Establishment and analysis of serum two-dimensional gel electrophoresis profiles of myasthenia gravis patients with spleen and kidney deficiency syndrome Zhong Xi Yi Jie He Xue Bao 2007 5 150 154 17352870 10.3736/jcim20070210

[B46] Cheng C Wu G Yeung SC Li R Bella AE Pang J Zhong FT Luo H Jin Y Pan J Serum protein profiles in myasthenia gravis Ann Thorac Surg 2009 88 1118 1123 19766792 10.1016/j.athoracsur.2009.05.032

[B47] Lu YH Wang CM Chen ZX Zhao H Chen JY Liu XB Zhao H Kwan YW Lin HQ Ngai SM Serum metabolomics for the diagnosis and classification of myasthenia gravis Metabolomicsin press

[B48] Osserman KE Genkins G Studies in myasthenia gravis: review of a twenty-year experience in over 1200 patients The Mount Sinai Journal of Medicine, New York 1971 38 497 537 4941403

[B49] Liu J Zhu P Peng J Li K Du J Gu J Ou Y Identification of disease-associated proteins by proteomic approach in ankylosing spondylitis Biochem Biophys Res Commun 2007 357 531 536 17434140 10.1016/j.bbrc.2007.03.179

[B50] Mackiewicz A Khan MA Reynolds TL van der Linden S Kushner I Serum IgA, acute phase proteins, and glycosylation of alpha 1-acid glycoprotein in ankylosing spondylitis Ann Rheum Dis 1989 48 99 103 2467628 10.1136/ard.48.2.99 PMC1003692

[B51] Sottrup-Jensen L Alpha-macroglobulins: structure, shape, and mechanism of proteinase complex formation J Biol Chem 1989 264 11539 11542 2473064

[B52] Howard FM Silverstein MN Mulder DW The coexistence of myasthenia gravis and pernicious anemia Am J Med Sci 1965 250 518 526 5846032 10.1097/00000441-196511000-00005

[B53] Harrison RA Farries TC Northrop FD Lachmann PJ Davis AE Structure of C3f, a small peptide specifically released during inactivation of the third component of complement Complement 1988 5 27 32 3338271 10.1159/000463028

[B54] Liu A Lin H Liu Y Cao X Wang X Li Z Correlation of C3 level with severity of generalized myasthenia gravis Muscle Nerve 2009 40 801 808 19670317 10.1002/mus.21398

[B55] Tüzün E Scott BG Goluszko E Higgs S Christadoss P Genetic evidence for involvement of classical complement pathway in induction of experimental autoimmune myasthenia gravis J Immunol 2003 171 3847 3854 14500686 10.4049/jimmunol.171.7.3847

[B56] Thielens NM Colomb MG A model system for the study of the assembly and regulation of human complement C3 convertase (classical pathway) Eur J Immunol 1986 16 617 622 3487454 10.1002/eji.1830160606

[B57] Soltys J Kusner LL Young A Richmonds C Hatala D Gong B Shanmugavel V Kaminski HJ Novel complement inhibitor limits severity of experimentally myasthenia gravis Ann Neurol 2009 65 67 75 19194881 10.1002/ana.21536 PMC3045826

[B58] Hilgenfeldt U Linke R Riester U König W Breipohl G Strategy of measuring bradykinin and kallidin and their concentration in plasma and urine Anal Biochem 1995 228 35 41 8572285 10.1006/abio.1995.1311

[B59] Bhat R Axtell R Mitra A Miranda M Lock C Tsien RW Steinman L Inhibitory role for GABA in autoimmune inflammation Proc Natl Acad Sci USA 2010 107 2580 2585 20133656 10.1073/pnas.0915139107 PMC2823917

[B60] Kelley JM Hughes LB Bridges SL Does gamma-aminobutyric acid (GABA) influence the development of chronic inflammation in rheumatoid arthritis? J Neuroinflammation 2008 5 1 18171484 10.1186/1742-2094-5-1 PMC2235846

[B61] Ernster L Dallner G Biochemical, physiological and medical aspects of ubiquinone function Biochim Biophys Acta 1995 1271 195 204 7599208 10.1016/0925-4439(95)00028-3

[B62] Schmelzer C Lindner I Rimbach G Niklowitz P Menke T Döring F Functions of coenzyme Q10 in inflammation and gene expression Biofactors 2008 32 179 183 19096114 10.1002/biof.5520320121

[B63] Kim S Hong I Hwang JS Choi JK Rho HS Kim DH Chang I Lee SH Lee MO Hwang JS Phytosphingosine stimulates the differentiation of human keratinocytes and inhibits TPA-induced inflammatory epidermal hyperplasia in hairless mouse skin Mol Med 2006 12 17 24 16838068 10.2119/2006-00001.Kim PMC1514555

[B64] Shimada H Rajagopalan LE Rho-kinase mediates lysophosphatidic acid-induced IL-8 and MCP-1 production via p38 and JNK pathways in human endothelial cells FEBS Lett 2010 584 2827 2832 20434448 10.1016/j.febslet.2010.04.064

